# A reliable tool for assessment of acceptance of e-consultation service in hospitals: the modified e-consultation Technology Acceptance Model (TAM) questionnaire

**DOI:** 10.1186/s42506-025-00187-x

**Published:** 2025-04-22

**Authors:** Rasha Ashmawy, Sally Zeina, Ehab Kamal, Khaled Shelbaya, Nermeen Gawish, Sandy Sharaf, Elrashdy M. Redwan, Azza Mehanna

**Affiliations:** 1 Department of Clinical Research, Maamora Chest Hospital, Alexandria, MoHP Egypt; 2 Clinical Research Administration, Directorate of Health Affairs, Alexandria, MoHP Egypt; 3 Medical Research Division, National Research Center, Giza, Egypt; 4 National Heart Institute, Giza, Egypt; 5 Research Department, Alnas Hospital, Cairo, Egypt; 6 Minister’s Technical Office, MoHP, Cairo, Egypt; 7 Department of Biological Science, Faculty of Science, King Abdulaziz University, Jeddah, Saudi Arabia; 8 Protein Research Department, Genetic Engineering and Biotechnology Research Institute, City of Scientific Research and Technological Applications (SRTA-City), New Borg EL-Arab, Alexandria, Egypt; 9 Department of Administration and Behavioral Sciences, High Institute of Public Health, Alexandria University, Alexandria, Egypt

**Keywords:** E-consultation, Questionnaire validation, Telehealth, Technology acceptance model

## Abstract

**Introduction:**

Innovative approaches like e-consultation services are critical for improving access to healthcare and promoting equity, particularly in under-resourced settings. Despite their growing prominence, limited tools are available to assess healthcare professionals’ acceptance and satisfaction with these services. This study aimed to validate the modified e-consultation TAM questionnaire as a reliable instrument for assessing physicians’ perspectives on e-consultation service.

**Methods:**

This study focuses exclusively on physicians receiving (not providing) e-consultation service within the Egyptian Ministry of Health and Population. The tool used for assessing their acceptance of the service consists of three sections: demographic data, items assessing perceived usefulness (PU) and perceived ease of use (PEU) of e-consultation, and questions addressing physicians’ satisfaction, challenges, and suggestions to improve e-consultation services. The questionnaire was subjected to thorough validation, including face validity evaluated by an expert panel and construct validity which was assessed through factor analysis.

**Results:**

The modified e-consultation TAM questionnaire demonstrated excellent internal reliability, with Cronbach’s alpha coefficient exceeding 0.92 for both PU and PEU. Exploratory factor analysis identified two domains, PU and PEU, explaining 81.17% of the variance, with factor loadings ranging from 0.661 to 0.912. Confirmatory factor analysis (CFA) confirmed the two-factor model, with standardized factor loadings between 0.80 and 0.95, a Comparative Fit Index (CFI) of 0.95, and a Root Mean Square Error of Approximation (RMSEA) of 0.084.

**Conclusion:**

The modified e-consultation TAM questionnaire proves to be a reliable and valid tool for evaluating physicians’ acceptance of and satisfaction with e-consultation service. This tool offers potential for future research and practical applications, providing valuable insights to improve the implementation of e-consultation services and inform strategies for advancing healthcare access and equity globally.

**Supplementary Information:**

The online version contains supplementary material available at 10.1186/s42506-025-00187-x.

## Introduction

Health systems continuously strive to optimize access to healthcare services and adopt innovative approaches to improve service accessibility and promote health equity. One such approach is the implementation of diverse care delivery models, including e-consultation services [1]. The World Health Organization (WHO) described e-consultation as a cost-effective and secure tool that facilitates the delivery of healthcare services, education, and research [2]. In Egypt, along with several other countries including India, China, and Saudi Arabia [3–5], e-consultation was introduced on a limited scale as an effective means of exchanging health information between healthcare providers and obtaining consultations from specialists [6]. This innovation has the potential to increase healthcare access and equity, especially in rural or remote areas where specialist care is scarce [7].

Since 2020, the COVID-19 pandemic has significantly influenced healthcare systems worldwide, necessitating the adoption of remote communication and virtual consultations among healthcare specialists to ensure the delivery of adequate healthcare services [8]. During the COVID-19 pandemic, physicians utilized e-consultation services in quarantine hospitals to provide multidisciplinary care not only for COVID-19 patients but also for other patients due to the closure of hospitals during the pandemic. This shift in healthcare service delivery was observed globally. This experience not only enhanced their ability to deliver high-quality patient care but also significantly reduced the risk of infection [9]. In Egypt, following the pandemic, data extracted from the e-consultation system provided by the Ministry of Health and Population (MoHP) indicated the need to optimize the benefits of this service. In response, the MoHP aimed to enhance capacity by increasing the number of e-consultation units from 150 to 300 across 26 governorates. By the end of 2022, healthcare providers (HCPs) in the Egyptian governmental sector had conducted over 27,000 e-consultations, with the most requested being dermatology, internal medicine, and pediatrics, to improve healthcare delivery[10]. Despite the growing use of e-consultation services among Egyptian HCPs, there is limited understanding of their experiences, particularly regarding acceptance and satisfaction with these services [11].

Several studies have assessed physicians’ acceptance of telehealth systems in Arab countries, most rely on the Technology Acceptance Model (TAM) [12–14]. TAM emphasizes perceived usefulness (PU) and perceived ease of use (PEU) as key factors influencing technology acceptance [15, 16]. This study aimed to modify TAM and validate the modified version to assess the acceptance and satisfaction with e-consultation service among physicians. By providing a validated tool, the findings may support the optimization of e-consultation systems, contributing to enhanced healthcare delivery and improved health equity, particularly in underserved areas.

## Methods

### Modification and pretesting of the e-consultation Technology Acceptance Model (modified e-consultation TAM)

The study included four following phases:

- Phase 1: Arabic translation and adaptation of the TAM questionnaire.
- Phase 2: Pre-testing of the modified e-consultation TAM.
- Phase 3: Investigation of the validity and reliability of the modified e-consultation TAM
- Phase 4: Translation of the modified e-consultation TAM into English.

#### Phase 1: Arabic translation and adaptation of the TAM questionnaire

Two steps were conducted to achieve this phase:

We reviewed the literature for validated questionnaires assessing physicians’ acceptance of e-consultation technology[17–19]. One of the few tools identified was the original TAM questionnaire developed by Davis [15]. According to Lewis [20], four different versions of the original TAM exist in the literature, varying in the response (using either numbers or labels) and the order in which responses are presented, which can increase either from left to right or right to left [20]. We selected version 4 as the most suitable, employing a 7-point Likert scale where agreement increased from left to right, and aligns with the typical scale preferences in many cultural contexts, including those of the target population. This version also assesses both current and potential users of the technology. Participants indicated their responses on a scale, ranging from 1 to 7. We modified the TAM questionnaire (version 4) and translated it into Arabic to establish a new tool (the modified e-consultation TAM) that is more relevant to e-consultation and the target population (physicians receiving e-consultation). This modification also allowed us to collect additional data by adding a new section to the questionnaire, which focused on participants’ satisfaction with the e-consultation service, challenges encountered in its use, and suggestions for improvement.

The final study questionnaire included 23 items as a total: 8 items for sociodemographic characteristics, 12 items from the modified TAM (covering PU and PEU), and 3 additional questions: one close-ended question (yes/no) for overall satisfaction with the service, one open-ended question about challenges encountered during e-consultation, and a final open-ended question for suggestions to enhance the service.

#### Phase 2: Pre-testing of the modified e-consultation TAM questionnaire (Arabic version)

Pre-testing was conducted using a sample of 17 physicians working at a governmental hospital receiving e-consultation in Alexandria. Most participants were females (70.6%), with a median age of 43 years (ranging from 38 to 55) and a median of 13 years of work experience. Additionally, 94.1% of the participants resided in urban areas. A 23-item paper questionnaire was administered, taking an average of ten minutes to complete. Participants provided feedback on clarity, content, appropriateness, and format, leading to several modifications. Specific changes in wording and syntax were performed. These modifications were incorporated into the final version of the questionnaire used for the larger validation phase.

#### Phase 3: Investigation of the validity and reliability of the modified e-consultation TAM questionnaire (Arabic version) using a survey.

To ensure the face and content validity of the modified e-consultation TAM questionnaire, a systematic evaluation was conducted by a panel of experts. The experts assessed the clarity, relevance, cultural appropriateness, and comprehensiveness of the items to ensure they accurately represented the intended constructs within the Egyptian healthcare context. Five iterative rounds of review and discussion were conducted, during which the panel provided feedback and suggested modifications until a consensus was reached that no further changes were necessary. The Arabic version of the questionnaire was refined by professionals with expertise in healthcare and Arabic linguistics to ensure better readability and cultural alignment. The expert panel, which included specialists in public health, behavioral sciences, and e-consultation, was formally acknowledged in the declaration section for their valuable contributions.

#### Phase 4: Translation of the modified e-consultation TAM into English

The Arabic-modified e-consultation TAM questionnaire was translated into English by two independent professional translators fluent in Arabic and English. Supplementary Table S2 presents discrepancies between the original TAM 4 and the translated modified e-consultation TAM.

### Study design and setting

A cross-sectional anonymous survey was conducted across 42 governmental hospitals under the MoHP, spanning 16 governorates in Egypt. Physicians receiving (not providing) e-consultation at these hospitals were invited to participate via WhatsApp groups dedicated to MoHP hospitals. Participants completed an online questionnaire (Google Form) over two weeks, from January 30 to February 12, 2023. The Google Form, created by MoHP, utilized SSL encryption to protect data during transmission and maintain participant anonymity. Access to the collected data was restricted to authorized research team members only, and the data were stored in a secure Google Drive folder with controlled permissions. The survey did not request personally identifiable information (PII), and any potentially identifying data were anonymized. Data were retained only for the duration of the study and securely deleted once the analysis was completed.

### Study population

*Inclusion criteria:* Physicians with at least a year of experience at MoHP hospitals and using the “telemedicine platform” for expert consultation.

*Exclusion criteria:* Academic consultants providing e-consultation at MoHP hospitals and those unwilling to participate in the study.

### Sample size and sampling technique

A convenient sample of 120 physicians receiving e-consultation at governmental hospitals was included. The sample size was calculated based on the rule of 3–20 observations per item, with 100 being considered optimal funding [21]. In line with commonly accepted practices, we used the rule of thumb of 10 participants per item.

Participant recruitment employed snowball sampling. Initially, participants were selected and invited to complete the survey after obtaining formal permission from the hospital administration. These initial participants were then asked to forward the survey link to eligible colleagues within their network who were using the e-consultation service but were not present in the formal WhatsApp groups. This approach facilitated reaching a broader range of respondents and ensured a diverse sample from various specialties and governorates.

### Instrument

We used the finalized version of the instrument, comprising 23 items distributed over three sections, after pretesting and adaptation.

### Overview of the e-consultation process in the MoHP

#### Platform

The MoHP utilized a dedicated “Telemedicine Platform” for secure video consultations between physicians.

#### Consultation structure

Each e-consultation session was scheduled for approximately 20 to 30 min, allowing for case presentation and discussion.

#### Case selection

Consulting physicians selected cases that were sufficiently complex or when a specialist was unavailable at their hospital, such as a chest hospital needing input in gynecology, cardiology, or oncology.

#### Documentation

Before the e-consultation, consulting physicians prepared a brief case summary of the health record. IT specialists ensured all relevant patient documents were uploaded to the platform for consultant access.

#### Follow-up

After each e-consultation, the consulting specialist provided written recommendations, which were incorporated into the patient’s health record and updated on the platform to indicate case status—whether completed, requiring further investigation, or needing transfer to another hospital. A team at MoHP facilitated the execution of the consultant’s recommendations.

#### Training

Before implementation, the MoHP conducted training programs for all participating physicians, IT personnel, and coordinators on using the e-consultation platform and best practices for remote consultation. Ongoing training was also provided for all new members.

### Statistical analysis

Data were managed and missing or incomplete responses were handled using listwise deletion, excluding participants with incomplete responses to maintain the integrity of the factor analysis and avoid potential bias from imputing missing data. We used various statistical techniques to analyze the data, including mean and standard deviation for continuous data and counts and percentages for categorical data, providing a comprehensive overview of participants’ characteristics. To refine the factor structure, we used the dataset for Exploratory Factor Analysis (EFA) with maximum likelihood extraction. Principal Component Analysis (PCA) was also performed with Varimax rotation and Kaiser normalization to explore the questionnaire’s factorial structure as independent factors [22]. We assessed factor loadings, eigenvalues, and scree plots, considering items with loadings of at least 0.50 as acceptable.

To evaluate the goodness of fit between the identified factor structure from EFA and the observed data, we conducted Confirmatory Factor Analysis (CFA), a type of Structural Equation Modeling (SEM). SEM was further employed to assess the convergent and discriminant validity of the constructs and model fit metrics. Our criteria for evaluating model fit were a Comparative Fit Index (CFI) greater than 0.9 and a Root Mean Square Error of Approximation (RMSEA) equal to 0.08. Additionally, we examined the reliability of items within each factor using Cronbach’s alpha coefficient. The software used for CFA was R version 4.2.1, while EFA and other analyses were conducted using SPSS Statistics 25 (SPSS Inc., Chicago, IL, USA) [23].

The necessary assumptions for exploratory factor analysis (EFA) and confirmatory factor analysis (CFA) were tested and reported. Normality was assessed using Kolmogorov–Smirnov Test to ensure the data distribution met the assumptions for factor analysis. Sampling adequacy was evaluated using the Kaiser–Meyer–Olkin (KMO) test, which yielded a value greater than 0.6, indicating that the sample size was sufficient for EFA. Additionally, linearity between variables was checked to ensure the validity of the factor models. These assumptions were confirmed to be met, supporting the robustness of the analysis.

## Results

### Characteristics of the respondents for construct validity and reliability

Table 1 displays the demographic characteristics of the respondents. Within 2 weeks during which the questionnaire was accessible to e-consultation users across Egypt, 120 physicians responded. The respondents had a mean age of 37.3 ± 7.6 years. Of these respondents, about half (51.67%) were female, 55.83% were specialists, and 85.83% resided in urban areas. The median work experience was 10 years, ranging from 1 to 32 years. Respondents’ affiliations were distributed as follows: 76.67% from general hospitals, 11.66% from tropical and fevers hospitals, 7.50% from pulmonary disease hospitals, 2.50% from pediatric hospitals, and 1.67% from ophthalmology hospitals.

**Table 1:** Demographics and characteristics of questionnaire respondents (Physicians, Egypt, 2023)

Characteristics (*N* = 120)	No.	%
Gender (female)	62	51.67
Age (mean ± SD), years	37.3 ± 7.6	
Years of experience [median (min–max)]	10 (1–32)	
Position		
General practitioner	4	3.34
Resident	39	32.50
Specialist	67	55.83
Consultant	10	8.33
Hospital area (urban)	103	85.83
Hospital type		
General	92	76.67
Tropical and fevers	14	11.66
Pulmonary diseases	9	7.50
Pediatric	3	2.50
Ophthalmology	2	1.67
Period using telemedicine		
Less than 1 month	13	10.83
1–3 months	45	37.50
4–6 months	37	30.84
7–9 months	11	9.17
10–12 months	4	3.33
More than 1 year	10	8.33

**Fig. 1:**
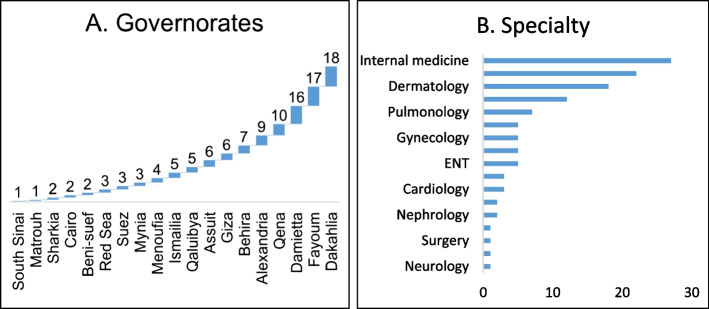
**A** Distribution of participating physicians by governorate of their hospitals (Egypt, 2024). **B** Distribution of participating physicians by specialty (Egypt, 2023)

Figure 1 shows physicians’ specialties and governmental distribution around Egypt. Panel A shows the number of physicians from each governorate, with the highest representation from South Sinai (18 physicians) and the lowest from the Red Sea (1 physician). Other governorates, such as Alexandria (16 physicians) and Daqahliya (5 physicians), have moderate representation. Panel B displays the distribution of specialties, with Internal Medicine (27 physicians) and Pediatrics (18 physicians) being the most common. Other specialties include 7 in Dermatology, 6 in Pulmonology, and 5 in ICU, with a smaller representation in specialties like Neurology, which had only one physician.

### Internal consistency and questionnaire adequacy

The summary statistics of the modified e-consultation TAM questionnaire show that all items have mean scores above 5, indicating generally positive responses, with Q12 having the highest mean of 5.92. The standard deviations are relatively consistent, indicating a moderate spread of responses around the mean. Skewness and kurtosis values suggest a tendency towards higher ratings. The total score of PU and PEU indicates that both constructs are generally rated positively, with mean scores of 42.18 ± 12.8 for PU, and 23.1 ± 5.18 for PEU. The skewness and kurtosis values suggest a negatively skewed distribution for both constructs, with PEU showing higher kurtosis. For PU, 3.2% of participants rated the minimum score of 8, and 15.1% rated the maximum score of 56. For PEU, 0.8% of participants rated the minimum score of 4, and 26.2% rated the maximum score of 28, supplementary table S3.

Cronbach’s alpha is a widely used method for measuring internal consistency, and for the TAM, its factor Cronbach’s alpha was 0.95. Before conducting an exploratory factor analysis (EFA), we assessed the appropriateness of the sample and the assumptions of sphericity. Based on the KMO measure, which was very high at 0.925, our sample of 120 respondents was deemed suitable for conducting EFA. Additionally, Bartlett’s test of sphericity rejected the null hypothesis that the correlation matrix was identical with a significance level of less than 0.001. Therefore, we met all the necessary assumptions for conducting EFA.

### Construct validity

#### Exploratory factor analysis (EFA)

**Fig. 2:**
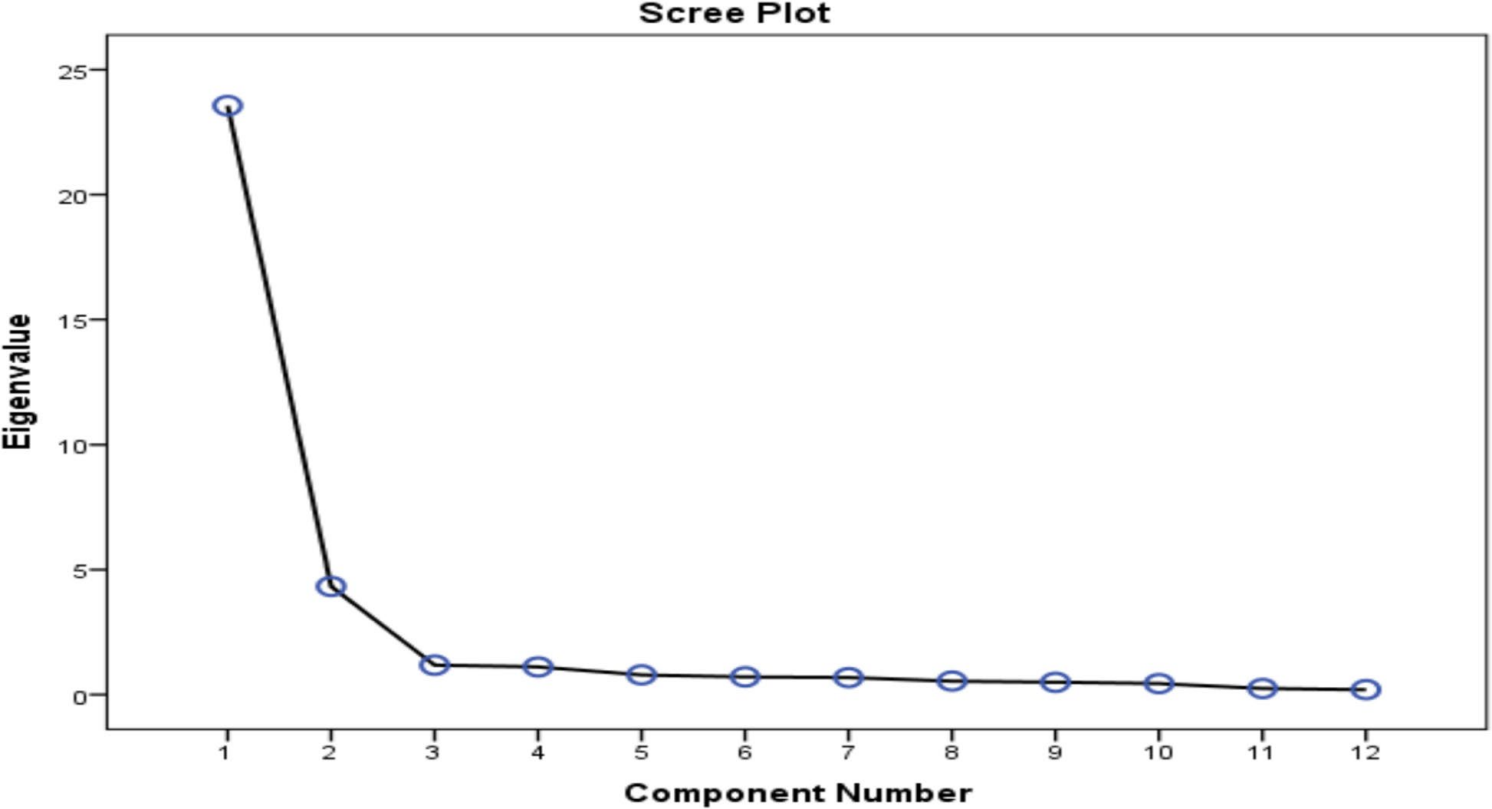
Scree plot displays the eigenvalues

Table 2 presents the correlation matrix of the modified e-consultation TAM questionnaire, which ranged from 0.321 to 0.855 and had diagonal symmetry. After extracting the commonalities, the values ranged from 0.736 to 0.882, all of which were above 0.5, indicating that no item needed to be removed from the TAM tool. To determine the number of factors to extract above the intersection point, we used both the Kaiser Criterion and the scree plot. As shown in Fig. 2, we found that the two-factor solution was the best for the modified e-consultation TAM questionnaire.

**Table 2:** Correlation matrix of the modified e-consultation TAM questionnaire items

	**Q1**	**Q2**	**Q3**	**Q4**	**Q5**	**Q6**	**Q7**	**Q8**	**Q9**	**Q10**	**Q11**	**Q12**
Q1	1	.809^**^	.771^**^	.722^**^	.807^**^	.772^**^	.321^**^	.808^**^	.618^**^	.456^**^	.364^**^	.377^**^
Q2	.809^**^	1	.760^**^	.753^**^	.776^**^	.792^**^	.374^**^	.778^**^	.673^**^	.518^**^	.426^**^	.477^**^
Q3	.771^**^	.760^**^	1	.741^**^	.812^**^	.818^**^	.378^**^	.775^**^	.603^**^	.408^**^	.346^**^	.408^**^
Q4	.722^**^	.753^**^	.741^**^	1	.797^**^	.855^**^	.474^**^	.757^**^	.780^**^	.566^**^	.445^**^	.578^**^
Q5	.807^**^	.776^**^	.812^**^	.797^**^	1	.912^**^	.420^**^	.804^**^	.719^**^	.507^**^	.430^**^	.495^**^
Q6	.772^**^	.792^**^	.818^**^	.855^**^	.912^**^	1	.467^**^	.793^**^	.771^**^	.521^**^	.444^**^	.514^**^
Q7	.321^**^	.374^**^	.378^**^	.474^**^	.420^**^	.467^**^	1	.427^**^	.568^**^	.597^**^	.761^**^	.734^**^
Q8	.808^**^	.778^**^	.775^**^	.757^**^	.804^**^	.793^**^	.427^**^	1	.724^**^	.541^**^	.444^**^	.519^**^
Q9	.618^**^	.673^**^	.603^**^	.780^**^	.719^**^	.771^**^	.568^**^	.724^**^	1	.658^**^	.591^**^	.635^**^
Q10	.456^**^	.518^**^	.408^**^	.566^**^	.507^**^	.521^**^	.597^**^	.541^**^	.658^**^	1	.724^**^	.768^**^
Q11	.364^**^	.426^**^	.346^**^	.445^**^	.430^**^	.444^**^	.761^**^	.444^**^	.591^**^	.724^**^	1	.854^**^
Q12	.377^**^	.477^**^	.408^**^	.578^**^	.495^**^	.514^**^	.734^**^	.519^**^	.635^**^	.768^**^	.854^**^	1

^**^Person correlation is significant at the 0.01 level (2-tailed), *n* = 120

Through EFA analysis of the resulting 12-item questionnaire, we identified two domains that accounted for 81.17% of the variance in the data. As outlined in Table 3, domain one (PU) was measured by eight items, while domain two (PEU) was measured by four items. The resulting domain distribution differed from the original TAM domain distribution. We validated the model by comparing the initial correlation matrix with the matrix generated from the latent variables to assess the quality of the obtained solution.

**Table 3:** Rotated Component Matrix using Varimax and Kaiser normalization method showing the distribution of items through the two TAM domains

	Component	
	**PU**	PEU
Q1	0.886	
Q2	0.851	
Q3	0.878	
Q4	0.813	
Q5	0.890	
Q6	0.886	
Q7		0.836
Q8	0.847	
Q9	0.661	
Q10		0.785
Q11		0.912
Q12		0.894

#### Confirmatory factor analysis (CFA)

**Fig. 3:**
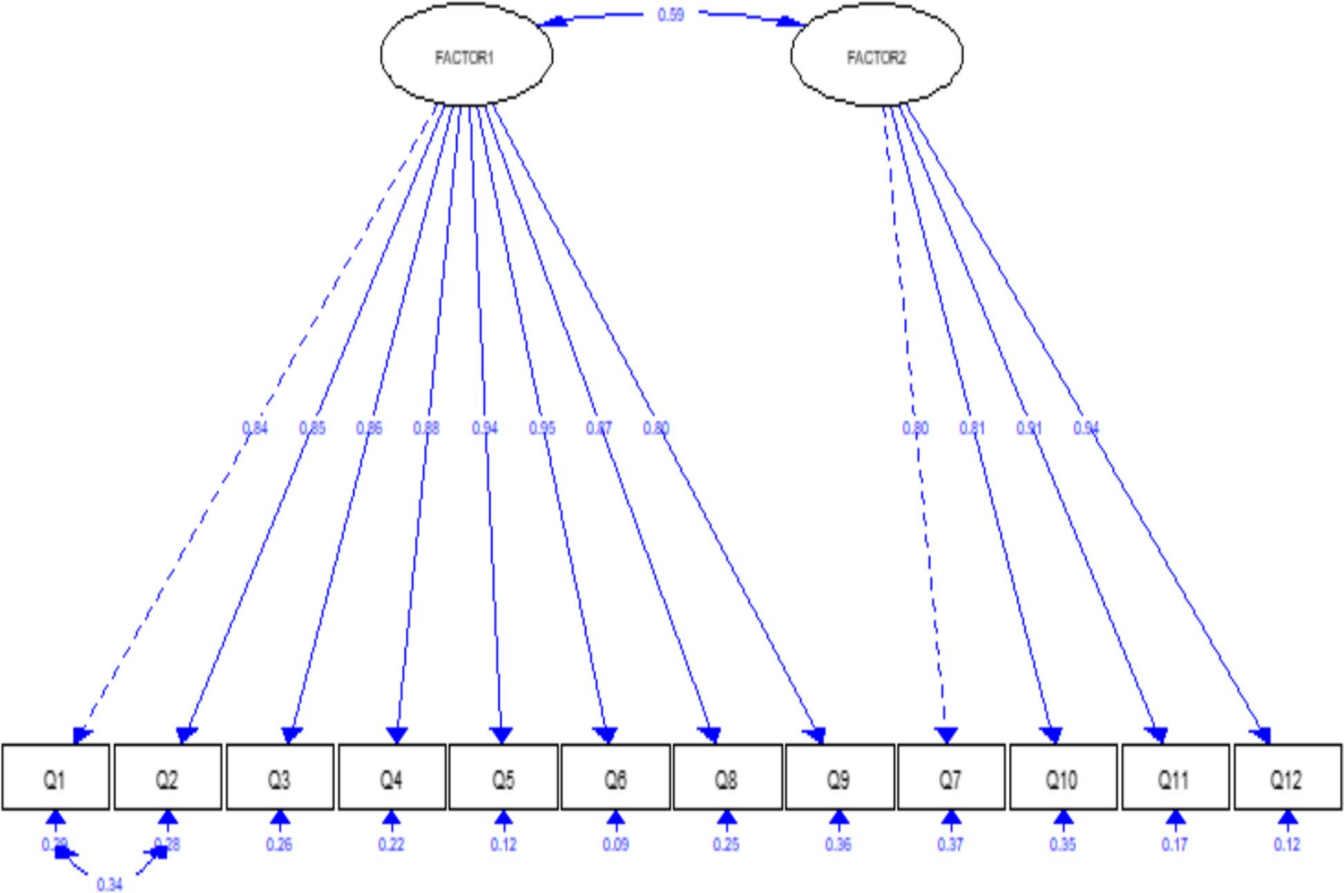
Confirmatory factor analysis of the modified e-consultation TAM questionnaire

We utilized the CFA to verify our hypothesis that the questionnaire consisted of two factors and to confirm that the 12 items were loaded under their respective factors, as identified in the EFA. Figure 3 illustrates the final CFA model using SEM. All loadings ranged from 0.80 to 0.95, and we obtained a CFI of 0.95 and an RMSEA of 0.084.

We recalculated the reliability for each domain of the modified e-consultation TAM, for PU (8 items) Cronbach’s alpha was 0.963, while for PEU (4 items) Cronbach’s alpha was 0.918.

### Bivariate analysis

Table S4 shows that PU and PEU have a significant positive correlation (*r* = 0.626, *p* < 0.001), while age and years of experience are strongly correlated (*r* = 0.896, *p* < 0.001). However, neither PU nor PEU shows a significant correlation with age or years of experience.

## Discussion

The results of this study demonstrate the utility of the modified e-consultation TAM in assessing physicians’ acceptance of the e-consultation service in Egypt. A previous study assessed the acceptance of telemedicine in Egypt in 2020; however, the questionnaire used in this study was directed to the general population, not the physicians. It showed that most Egyptians considered telemedicine’s advantages favorably [24]. Several questionnaires were designed for healthcare workers in Arab countries; however, the scope of telemedicine was broad and included communication with patients, rather than being focused on e-consultations among physicians [19, 25]. In this study, the fourth version of the original TAM was modified to target e-consultation particularly among physicians. Furthermore, this modified e-consultation TAM version increases the likelihood of better response quality by incorporating a relatively short list of items (23 items) [26].

The demographic characteristics of the respondents indicate a diverse sample of physicians regarding age, gender, specialization, years of experience, and urban residence. It is important to consider these demographic factors when interpreting the study findings, as they may influence physicians’ perceptions and acceptance of e-consultation services. The inclusion of the satisfaction, suggestions, and challenges section in the modified e-consultation TAM questionnaire is a significant addition to the study. This section provides deeper insights into the factors that influence physicians’ acceptance of or resistance to e-consultation technology. By exploring these factors, the study sheds light on potential challenges that may hinder technology adoption and identifies areas for improvement. This information can guide healthcare organizations in developing targeted interventions and strategies to address the identified challenges and promote the successful implementation of e-consultation services [27].

The internal consistency assesses the relationship between the questionnaire components. It represents an easy method to check the reliability during the questionnaire development process as it does not require a comparative repeating of the questions [28]. The internal consistency of the modified e-consultation TAM questionnaire and its two factorial components, namely, PU and PEU, showed Cronbach’s alpha coefficient of > 0.92, at least, indicating excellent internal reliability. This indicates that the items within the questionnaire are highly correlated and measure the same underlying construct, which is the acceptance of e-consultation service.

We utilized exploratory factor analysis to determine the number of hidden factors that could affect the physician’s acceptance, resulting in the identification of two factors that account for approximately 81% of the variance. These factors closely resemble the original TAM-4 model. We subsequently performed the maximum likelihood method to examine the loading pattern and ascertain the factor with the most significant influence on each variable. Following varimax rotation and the exclusion of commonality below 0.5, we observed that the following variables had the highest loadings on factor 1 (PU), indicating that this factor elucidates how e-consultation aids physicians in their duties and facilitates interaction with consultants for improved patient care outcomes: Q5 (0.890), Q3 (0.878), Q1, and Q6 (0.886), Q2 (0.851), Q8 (0.847), and Q4 (0.814). Conversely, Q9 (0.661) exhibited the lowest loading on factor 1. On the other hand, factor 2 (PEU) was primarily characterized by the following variables, which displayed the highest loadings: Q11 (0.912), Q12 (0.894), Q7 (0.836), and Q10 (0.785). This indicates that factor 2 elucidates how the ease of use of e-consultation affects physicians’ acceptance of this new technology. Thus, factor analyses, after Arabic translation and adaptation, revealed the shifting of two questions originally proposed for assessing PEU to PU. However, the performance of the two factors, even by using the confirmatory factor analysis, was quite assuring.

The Rotated Component Matrix further highlights the relationships between these factors and their associated variables. For PU, Q6 demonstrated the strongest association (0.890), reinforcing the system’s perceived usefulness in supporting physicians’ work, while Q9 had the lowest association (0.661), indicating that interaction clarity could be enhanced perhaps through giving more details or demonstrations of the management steps and techniques. Similarly, for PEU, Q11 had the highest loading (0.912), revealing that physicians perceive the system as easy to learn, whereas Q10 (0.785) indicated a need for enhancing the flexibility of the system. These results provide clear insights into what works well and what needs to be improved. However, lower scores for interaction clarity and flexibility point to areas that could be optimized. Focusing on these aspects while reinforcing the system’s strengths will help make the e-consultation service more effective and user-friendly.

Furthermore, The Comparative Fit Index (CFI) of 0.95 indicates an excellent fit of the model to the data, as it surpasses the commonly accepted threshold of 0.90 for a well-fitting model. Similarly, the RMSEA of 0.084, while slightly higher than the ideal value of 0.06, remains within the acceptable range (0.08), suggesting a reasonable model fit. These results collectively reinforce the validity and reliability of the modified instrument. By achieving high factor loadings (0.80–0.95) and strong internal consistency (Cronbach’s alpha of 0.963 for PU and 0.918 for PEU), the findings further validate the robustness of the instrument in measuring the intended constructs.

According to Hajesmaeel-Gohari et al. [18], the Telehealth Usability Questionnaire (TUQ), Telemedicine Satisfaction Questionnaire (TSQ), Service User Technology Acceptability Questionnaire (SUTAQ), Questionnaire for User Interaction Satisfaction (QUIS), System Usability Scale (SUS), Client Satisfaction Questionnaire (CSQ), Patient Satisfaction Questionnaire (PSQ), and TAM were the most common questionnaires for evaluating users’ satisfaction, usability, and acceptance of telemedicine technology. Its popularity is attributed to its simplicity, adaptability to diverse technologies and contexts, and robust theoretical foundation [18]. TAM is grounded in psychology and information systems theory, derived from the Theory of Reasoned Action (TRA). It is easy to understand and implement because it uses only a few core constructs to explain technology adoption. Moreover, it has been extensively tested and validated across numerous studies, proving its reliability and predictive power. Unlike fixed questionnaires like the SUS or CSQ, TAM provides a flexible framework. In addition, the CSQ, PSQ, and TUQ measure satisfaction but do not provide insights into technology acceptance [18, 29, 30].

TAM was first introduced by Davis [15] with its core constructs: Perceived Usefulness (PU), Perceived Ease of Use (PEU), and Behavioral Intention (BI) to use the system. It has evolved significantly since its development, with numerous modifications and extensions to address its limitations and adapt it to various contexts yielding TAM 2 (2000), Unified Theory of Acceptance and Use of Technology (UTAUT) (2003), and TAM 3 (2008) to incorporate user experience and psychological variables influencing PEU. While the original TAM focuses on the core PU and PEU constructs, TAM2, TAM3, and UTAUT expand to include social, cognitive, and situational factors. Later versions (TAM3, UTAUT) are more complex and comprehensive, suitable for diverse contexts [16, 27, 31, 32]. Likewise, Chau and Hu [33] proposed a three-dimensional model for telemedicine acceptance, including individual, organizational, and technological dimensions. However, this study focused on PU and PEU, the original constructs of Davis’s TAM [15, 33].

In the current study, we used the original TAM [15] version 4 after adapting it to target e-consultation among physicians in various areas of healthcare. Aiming to assess the physicians’ acceptance of the provided service and considering their time constraints and work overload, we settled on using the most simplified yet validated TAM (original TAM) after undergoing some adaptations to focus on the e-consultation service among physicians [15]. Of the four available formats of the original TAM, we decided to use version 4 as recommended by a study comparing the four versions based on the effect of response options order and labels. The study concluded that Version 4, with its numeric response options arranged with the magnitude of agreement increasing from left to right, appeared to be the best choice for most user research to reflect the true usability and collect experiential ratings rather than the likelihood of use [20].

A prior study by Gagnon et al. utilized a modified version of the TAM to assess healthcare professionals’ acceptance and adoption of a telemonitoring system designed for chronic care patients. The modified version extended the original TAM framework by integrating additional factors particularly relevant to healthcare settings including attitude, compatibility, subjective norm, facilitators, and habit. However, the questionnaire was much longer than the current modified e-consultation TAM (33 items), which increased the time and effort required for its completion, and did not specifically address e-consultation [27]. Another relevant questionnaire is the Perceived Telemedicine Importance, Disadvantages, and Barriers (PTIDB) questionnaire developed by Youssef et al. [17]. PTIDB included four domains targeting telemedicine in general: “the importance of using telemedicine,” “advantages of telemedicine,” “disadvantages of telemedicine,” and “barriers to utilizing telemedicine” [17]. In contrast, the currently modified e-consultation TAM addresses two domains: PU and PEU of e-consultation service in particular. These two domains shed light on physicians’ experience with e-consultation and whether they find it useful and easy to use and hence, draw a clear picture of their acceptance of this service.

The study findings highlight the value of the modified e-consultation TAM in identifying physicians’ acceptance of e-consultation service in Egypt through assessment of its perceived usefulness and perceived ease of use. Items of the questionnaire allow physicians to determine whether using e-consultation service enhanced the effectiveness and efficiency of the provided service and whether they were easily accommodated to its use. Two open-ended questions were added to the tool requiring participants to specify the challenges they face in utilizing the service and possible suggestions to mitigate these challenges. Physicians’ responses could pave the way for further research to elaborate on these challenges and limitations facilitating targeted strategies to boost e-consultation adoption and effectiveness.

### Study limitations and strengths

The strength of the modified e-consultation TAM questionnaire lies in its focused approach to telemedicine, specifically targeting e-consultation and its relatively concise and user-friendly questions. Our work confirms the accuracy and simplicity of the seven-point Likert rating scale employed in TAM (version 4), enhancing the utility of our tool [20, 34]. We tested the reliability and validity of the questionnaire by relying on the respondents’ practice-based perception of e-consultation techniques. The wide-scale implementation of e-consultation technology will provide an opportunity to validate further the perceptions gained through the modified e-consultation TAM questionnaire. This broader implementation will allow a more comprehensive assessment of the model’s findings and enhance our understanding of user acceptance in diverse healthcare settings. In addition to the English version, an Arabic version of the questionnaire is available as well, which enhances the feasibility of assessing the acceptability and satisfaction with e-consultation among physicians in other Arab countries.

Despite the previous points of strength, the questionnaire has some limitations. The third TAM theoretical dimension in the original conceptual framework model (behavioral intention) was not included in the questionnaire for simplicity as mentioned before. Moreover, the study participants were exclusively selected from the Egyptian MoHP hospitals, which may limit the generalizability of the study to other sectors in Egypt. Additionally, the questionnaire is a self-reporting tool, thus it is subjected to social desirability and information bias. Finally, the use of convenience and snowball sampling reduces the sample representativeness and limits the generalization of results.

## Conclusion and recommendations

Physicians’ perceptions of e-consultation services play a crucial role in the adoption and utilization of e-consultation services. Understanding these perceptions is essential for successful implementation and effective integration of e-consultation into healthcare systems. The modified e-consultation TAM provides a simple framework for assessing physicians’ acceptance of e-consultation service based on their perceived usefulness and perceived ease of use. Moreover, this validated questionnaire may provide insight into some of the possible challenges faced by physicians regarding the utilization of this technology, which may inspire future research to deliberate on these weaknesses and find potential solutions to them.

## Supplementary Information

Supplementary Material 1.

## Data Availability

Data will be available upon request from the corresponding author.

## Abbreviations

E-consultation: Electronic consultation
TAM: Technology Acceptance Model
WHO: World Health Organization
HCPs: Health Care Professionals
PU: Perceived usefulness
PEU: Perceived ease of use
MoHP: Ministry of Health and Population
SD: Standard deviation
EFA: Exploratory factor analysis
CFA: Confirmatory factor analysis
KMO: The Kaiser–Meyer–Olkin test
SEM: Structural equation modeling
CFI: Comparative Fit Index
RMSEA: Root Mean Square Error of Approximation
PCA: Principal component analysis
