# Supplementary material for: A reliable tool for assessment of acceptance of e-consultation service in hospitals: the modified e-consultation Technology Acceptance Model (TAM) questionnaire
Source: J Egypt Public Health Assoc. 2025 Apr 22;100:6. doi: 10.1186/s42506-025-00187-x (PMC12014876; doi:10.1186/s42506-025-00187-x)
Supplement: Supplementary file 1 — Supplementary Material 1. [file 42506_2025_187_MOESM1_ESM.docx]

**S1: Modified e- consultation TAM**

The research team extends the best wishes and invites you to participate in filling out this questionnaire, which aims to evaluate the physicians’ acceptance of the implemented e-consultation / telemedicine service in hospitals. Please, note that only physicians receiving the service are required to answer.

Your participation does not require you to reveal any personal information. Any information you provide will be solely used for research purposes. Filling out this questionnaire will take only a few minutes and your participation is entirely voluntary; you are completely free to withdraw at any time. Your participation is greatly appreciated.

- Write a code consisting of a letter and 3 numbers: ................................

A- Physicians' personal information:

1-Gender: Male Female

2- Age: .............

3- Job title: General Practitioner Resident Specialist Consultant

4- Specialty: .............................................. ...

5 - Number of years of experience in the specialty: ................................................

6- The hospital name: …………………………………

7 - The hospital location: Urban Rural

8- Governorate: ......................

**B- Evaluation of physicians’ acceptance of the application of e-consultation service:**

Kindly, read the following statements, then indicate the level of your agreement by choosing a number from 1 to 7 where, 1 represents “totally disagree” to the statement, and 7 represents “totally agree”.

1-Using e-consultation service helps me to quickly determine the diagnosis and treatment plan for the patient compared to traditional consultation (face-to-face).

1 2 3 4 5 6 7

2- Using e-consultation services improves the provision of health services to the patient.

1 2 3 4 5 6 7

3- Using e-consultation services in my work allows me to accurately diagnose a larger number of cases in the hospital.

1 2 3 4 5 6 7

4 - The use of the e-consultation service increases my efficiency and adds to my experience and knowledge.

1 2 3 4 5 6 7

5- Using the e-consultation service facilitates my work performance.

1 2 3 4 5 6 7

6- I found the use of e-consultation service useful in my job.

1 2 3 4 5 6 7

7- Learning the e-consultation technique is easy for me.

1 2 3 4 5 6 7

8 - The e-consultation technology allowed me to get the maximum possible benefit from the consultant physician.

1 2 3 4 5 6 7

9 - My interaction with e-consultation technology was clear and understandable.

1 2 3 4 5 6 7

10 - I found flexibility in interacting with the consultant physician through using e-consultation technology.

1 2 3 4 5 6 7

11 - It was easy for me to be proficient in using e-consultation technology.

1 2 3 4 5 6 7

12 - I found the e-consultation technology easy to use.

1 2 3 4 5 6 7

**C- Evaluation of physicians’ satisfaction, challenges, and suggestions for improvement in applying e-consultation service:**

1. What is your overall satisfaction regarding e-consultation service?

Yes No

1. If you faced any challenges while applying to the service, please mention: ....................................
2. If you have any suggestions to improve the service, please mention: …...............................................

**Table S2: Differences between TAM4 and Modified E-consultation TAM**

| **TAM4 questions** | **Modified E-consultation TAM** | **The change** |
| --- | --- | --- |
| 1. Using this product in my job enables me to accomplish tasks more quickly than other products in its class. | 1. Using e-consultation service helps to quickly determine the diagnosis and treatment plan for the patient compared to traditional consultation (face-to-face consultation). | Whole question. |
| 1. Using this product improves my job performance. | 1. Using e-consultation service improves the provision of health services to the patient. | Whole question. |
| 1. Using this product in my job increases my productivity. | 1. Using e-consultation services in my work allows me to accurately diagnose a larger number of cases in the hospital. | Whole question. |
| 1. Using this product enhances my effectiveness on the job. | 1. Using an e-consultation service increases my efficiency and adds to my experience and knowledge. | - “e-consultation service” instead of “this product”.  - “adds to my experience and knowledge” is added. |
| 1. Using this product makes it easier to do my job. | 1. Using the e-consultation service facilitates my work performance. | Whole question. |
| 1. I have found this product useful in my job. | 1. I find the use of e-consultation services useful in my job. | “e-consultation service’ instead of “this product”. |
| 1. Learning to operate this product was easy for me. | 1. Learning the e-consultation technique is easy for me. | “the e-consultation technique” instead of “to operate this product”. |
| 1. I found it easy to get this product to do what I want it to do. | 1. The e-consultation technology allowed me to get the maximum possible benefit from the consultant physician. | Whole question. |
| 1. My interaction with this product has been clear and understandable. | 1. My interaction with the e-consultation technology was clear and understandable. | “e-consultation service” instead of “this product”. |
| 1. I found this product to be flexible to interact with. | 1. I found flexibility in interacting with the consultant physician through using e-consultation technology. | the whole question. |
| 1. It was easy for me to become skillful at using this product. | 1. It was easy for me to become proficient in using e-consultation technology. | “e-consultation service” instead of “this product”. |
| 1. I found this product easy to use. | 1. I found the e-consultation technology easy to use. | “e-consultation service” instead of “this product”. |
| **Two sections were added:**   1. Demographics 2. physicians’ satisfaction, challenges, and suggestions for improvement in applying e-consultation service. | | |

**Table S3: Summary Statistics of Modified e-Consultation TAM Questionnaire Data**

| **Item** | **Mean ± SD** | **Min** | **Max** | **IQR** | **Skewness** | **Kurtosis** |
| --- | --- | --- | --- | --- | --- | --- |
| Q1 | 5.08 ± 1.7 | 1 | 7 | 3 | -0.68 | -0.27 |
| Q2 | 5.02 ± 1.9 | 1 | 7 | 3 | -0.93 | -0.12 |
| Q3 | 5.03 ± 1.9 | 1 | 7 | 3 | -0.83 | -0.35 |
| Q4 | 5.65 ± 1.7 | 1 | 7 | 2 | -1.33 | 1.04 |
| Q5 | 5.13 ± 1.8 | 1 | 7 | 3 | -0.82 | 0.20 |
| Q6 | 5.29 ± 1.8 | 1 | 7 | 3 | -0.91 | 0.20 |
| Q8 | 5.19 ± 1.9 | 1 | 7 | 3 | -0.95 | -0.05 |
| Q9 | 5.62 ± 1.6 | 1 | 7 | 2 | -1.36 | 1.50 |
| **PU** | **42.18 ± 12.8** | **8 (3.2%)** | **56 (15.1%)** | **16.75** | **-1.07** | **0.61** |
| Q7 | 5.83 ± 1.4 | 1 | 7 | 2 | -1.23 | 0.86 |
| Q10 | 5.74 ± 1.5 | 1 | 7 | 2 | -1.44 | 1.94 |
| Q11 | 5.62 ± 1.5 | 1 | 7 | 2 | -1.26 | 1.43 |
| Q12 | 5.92 ± 1.3 | 1 | 7 | 2 | -1.58 | 2.93 |
| **PEU** | **23.1 ± 5.18** | **4 (0.8%)** | **28 (26.2%)** | **8** | **-1.41** | **2.32** |

**Table S4: Bivariate correlation (Spearmen’s correlation)**

|  | | | PU | PEU | Age | Years of experience |
| --- | --- | --- | --- | --- | --- | --- |
|  | PU | Correlation Coefficient | 1.000 | .626^**^ | .098 | .090 |
|  |  | Sig. (2-tailed) | . | .000 | .287 | .327 |
|  | PEU | Correlation Coefficient | .626^**^ | 1.000 | .102 | .112 |
|  |  | Sig. (2-tailed) | .000 | . | .269 | .225 |
|  | Age | Correlation Coefficient | .098 | .102 | 1.000 | .896^**^ |
|  |  | Sig. (2-tailed) | .287 | .269 | . | .000 |
|  | Years of experience | Correlation Coefficient | .090 | .112 | .896^**^ | 1.000 |
|  |  | Sig. (2-tailed) | .327 | .225 | .000 | . |
